# Demographic fluctuations in bloodstream *Staphylococcus aureus* lineages configure the mobile gene pool and antimicrobial resistance

**DOI:** 10.1038/s44259-024-00032-9

**Published:** 2024-05-07

**Authors:** Stephanie S. R. Souza, Joshua T. Smith, Michael M. Marcovici, Elissa M. Eckhardt, Nicole B. Hansel, Isabella W. Martin, Cheryl P. Andam

**Affiliations:** 1 Department of Biological Sciences, University at Albany, State University of New York, Albany, New York, NY USA; 2 Department of Molecular, Cellular and Biomedical Sciences, University of New Hampshire, Durham, NH USA; 3 Infectious Disease and Microbiome Program, Broad Institute of MIT and Harvard, Cambridge, MA USA; 4 Dartmouth-Hitchcock Medical Center and Dartmouth College Geisel School of Medicine, Lebanon, NH USA

**Keywords:** Pathogens, Molecular evolution

## Abstract

*Staphylococcus aureus* in the bloodstream causes high morbidity and mortality, exacerbated by the spread of multidrug-resistant and methicillin-resistant *S. aureus* (MRSA). We aimed to characterize the circulating lineages of *S. aureus* from bloodstream infections and the contribution of individual lineages to resistance over time. Here, we generated 852 high-quality short-read draft genome sequences of *S. aureus* isolates from patient blood cultures in a single hospital from 2010 to 2022. A total of 80 previously recognized sequence types (ST) and five major clonal complexes are present in the population. Two frequently detected lineages, ST5 and ST8 exhibited fluctuating demographic structures throughout their histories. The rise and fall in their population growth coincided with the acquisition of antimicrobial resistance, mobile genetic elements, and superantigen genes, thus shaping the accessory genome structure across the entire population. These results reflect undetected selective events and changing ecology of multidrug-resistant *S. aureus* in the bloodstream.

## Introduction

The Gram-positive opportunistic pathogen *Staphylococcus aureus* is a frequent colonizer of the skin, nasopharynx, and gastrointestinal tract^1^. Nasal carriage of *S. aureus* is common, with up to 30% of the human population asymptomatically and permanently colonized^2^. While most carriers of *S. aureus* do not develop illness, colonization often precedes infection, which can lead to a variety of debilitating and life-threatening diseases^3^. *S. aureus* can penetrate deep tissues and organs through localized skin infections or through the presence of prosthetic devices or indwelling catheters that provide a possible nidus for infection^4,5^. Because blood is normally a sterile environment, the presence of non-transient microbes in the bloodstream is a serious concern. *S. aureus* in the bloodstream causes high morbidity and mortality in the United States and worldwide^4,6^. Hospital mortality rates of patients with bloodstream infections due to *S. aureus* range between 15 and 40%^7,8^. *S. aureus* bacteremia increases the risk of complications such as infective endocarditis and metastatic infections, further exacerbating the disease burden and complicating effective treatment^9^. Multidrug-resistant and methicillin-resistant *S. aureus* (MRSA) can lead to higher mortality rates, persistent bacteremia, relapse, or therapeutic failure^10^. In the United States, a retrospective analysis of hospitalizations revealed that the odds of in-hospital death due to bacteremia was 15% greater for MRSA compared to methicillin-susceptible *S. aureus* (MSSA)^11^.

Long-term surveillance of *S. aureus*, and MRSA in particular, is critical to elucidating the genetic and ecological drivers of resistance and virulence, patterns of dissemination, and predicting future trends of disease. *S. aureus* comprises multiple genetically distinct clones, which vary in their phenotypic and epidemiological features, such as epidemic potential^12^, host association and specificity^13^, strategies to overcome host responses and infection outcomes^14,15^, and adhesion and biofilm formation^16^. Such variation is particularly critical in invasive disease. In a study of two major clonal complexes (CC), cytolytic toxicity and biofilm formation were reported to be predictive of a higher risk of mortality in infections caused by CC22 strains, but not those caused by CC30 strains^14^. It is, therefore, important to track the emergence, dissemination, and impact of specific high-risk clones in the population over the course of many years. Long-term studies allow the recognition of whether bloodstream infections and other invasive disease manifestations are caused by a newly emerged clone or a pre-existing minor strain that rose to prominence. Long-term studies are also important to clarify whether antimicrobial resistance (AMR) is caused by the expansion of a single clone, the replacement of different clones, and/or the horizontal transfer of resistance genes among different clones. Genomic epidemiology thus offers a powerful approach to pathogen surveillance: taking whole genome sequencing data of a pathogen to understand the distribution of a disease in a specified population, how the pathogen evolves, and which treatments and strategies may contain it^17^.

In this study, we aimed to characterize the circulating lineages of *S. aureus* from bloodstream infections and the contribution of individual lineages to resistance over time. We generated 852 high-quality short-read draft genome sequences of *S. aureus* isolates from patient blood cultures in a single hospital from 2010 to 2022. Our results revealed demographical fluctuations and evidence of recent replacement occurring between two high-frequency lineages, which were congruent with changes in AMR, mobile genetic elements, and virulence gene pools in the entire population. We discuss how long-term genomic surveillance is critical to understanding the epidemiological features of *S. aureus* clones, the ecology of resistance dissemination, and less common lineages that may act as reservoirs of AMR genes.

## Results

### S. aureus in the bloodstream is phylogenetically diverse, but a few lineages are present in high frequencies

We retrieved a total of 852 high-quality draft genomes of *S. aureus* isolates recovered from unique pediatric and adult patient blood cultures at Dartmouth-Hitchcock Medical Center (DHMC), New Hampshire, USA, sampled from December 2010 to February 2022 (Supplementary Figs. 1, 2). De novo genome assembly generated sequences of sizes ranging from 2.61 to 3.00 Mb (mean = 2.79 Mb) (Supplementary Table 1). The totality of genes in the population or pan-genome^18^ consisted of 4835 orthologous gene families. These can be classified into core genes (*n* = 2113 genes; present in 843–852 genomes), softcore genes (*n* = 15 genes; present in 809–842 genomes), shell genes (*n* = 866 genes; present in 127–808 genomes), and cloud genes (*n* = 1841; present in 1–126 genomes) (Supplementary Table 2). The average number of accessory genes per genome was 428 genes (range: 229–656 genes).

**Fig. 1:**
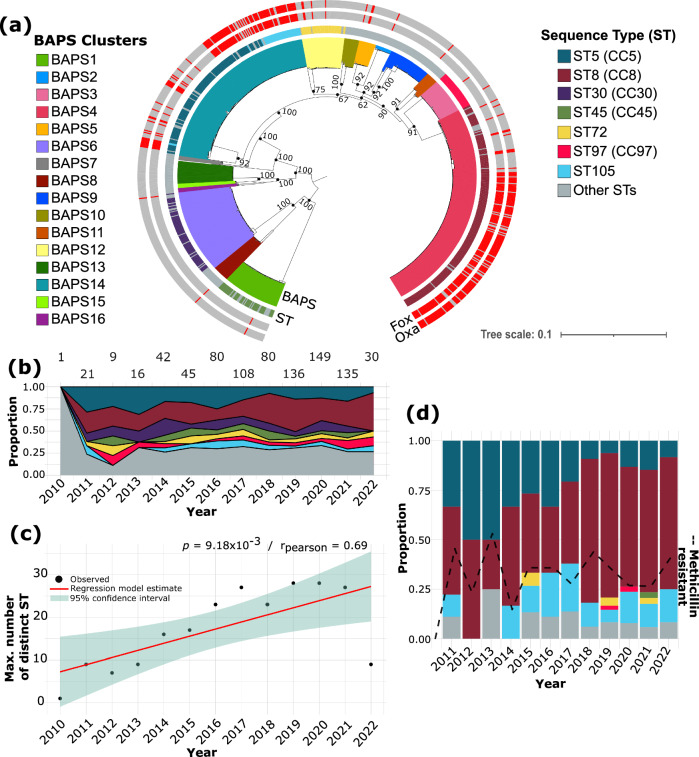
Phylogenetic relationships and in vitro methicillin resistance of 852 *S. aureus* isolates from bloodstream infection. **a** Midpoint-rooted maximum likelihood tree showing the phylogenetic relationships of 852 *S. aureus* genomes. The tree was built using 105,706 SNPs of aligned sequences of 2128 core genes. The tree scale represents the number of nucleotide substitutions per site. Support for phylogenetic nodes was estimated using 100 bootstrap replicates. For visual clarity, only the bootstrap values for major internal nodes are shown. For details of the bootstrapping analysis, the tree file is included in Supplementary Table 7. Colored lines extending out of the branches in the tree represent the 16 sequence clusters inferred by the program BAPS. The three outer rings represent the sequence type (ST) and phenotypic results of cefoxitin or oxacillin phenotypic screenings. Clonal complexes (CC) are shown in parentheses next to the STs in the color legend. **b** Yearly distribution of the proportion of major STs across the entire population throughout the study period. The number of isolates per year is shown at the top of the plot. **c** Linear regression model of the maximum number of different STs per year. **d** Yearly distribution of the number of isolates identified as methicillin-resistant based on in vitro testing as a proportion of the total number of isolates of each ST throughout the study period. The black dashed line shows the overall percentage of methicillin-resistant isolates over the years. For visual clarity, in panels **a**, **b**, **d**, only the seven most common STs are labeled, and less common STs were grouped together as Other STs.

Multi-locus sequence typing (MLST) analysis revealed the presence of 80 previously recognized sequence types (ST) (Fig. 1a and Supplementary Table 1). Seven STs (STs 5, 8, 30, 45, 72, 97, and 105) together comprised 70.1% (597/852) of the isolates in the population. Five STs represent major CCs recognized in *S. aureus*: ST5 (CC5), ST8 (CC8), ST30 (CC30), ST45 (CC45), and ST97 (CC97). The most prevalent STs were ST8 (225/852 genomes representing 26.4% of the population) and ST5 (132/852 genomes or 15.5%). A total of 74 isolates were identified as ST30, representing 8.7% of the population. ST45, ST72, ST97, and ST105 were each identified in ~5% of the isolates (ST45: 44/852 or 5.2%; ST72 and ST105: 41/852 each or 4.8%; ST97: 40/852 or 4.7%). We also identified 29 novel STs, which were all detected after 2017 (Supplementary Table 3). Only one isolate lacked a perfect match for one of the genes in the multi-locus sequence typing (MLST) scheme of *S. aureus*^19^. We identified 72 STs that were represented by a single isolate.

The maximum likelihood phylogenetic tree built from the sequence alignment of 105,706 SNPs of 2128 core + softcore genes revealed deep branching monophyletic lineages (Fig. 1a). These lineages corresponded to 16 sequence clusters inferred by Bayesian hierarchical clustering that groups together genetically similar sequences^20^. The largest sequence cluster BAPS4 contained 243 genomes representing nine STs (225 genomes from ST8, six genomes from ST1181, and 12 genomes from seven other STs, including a new ST first identified in this study). Another large cluster is BAPS14, which consisted of 197 genomes representing 19 STs (132 genomes from ST5, 41 genomes from ST105, and 24 genomes from 17 other STs, including three new STs first identified in this study). The third largest cluster BAPS6 (95 genomes), consisted mostly of ST30 genomes. BAPS3 was the only cluster consisting of genomes from a single ST (ST97). The majority of new STs belonged to BAPS6 (seven genomes) and BAPS1 (six genomes) (Supplementary Table 1).

The two high-frequency lineages, ST5 and ST8, were consistently present throughout the 12 years of this study (Fig. 1b). ST30 was present from 2011 until 2021, while STs 45, 72, 97, and 105 appeared at irregular intervals. Overall, we observed an increasing trend in the number of distinct STs over time (*p* = 9.18 × 10^−3^, *r*_Pearson_ = 0.69; Fig. 1c). However, this trend may be due to the increased number of isolates sampled, and the wider scope of communities from which isolates were obtained by DHMC during the latter years of our sampling period.

We next examined how the prevalence of methicillin resistance, defined as resistance to either cefoxitin and/or oxacillin and determined in vitro, has changed over time (Fig. 1d and Supplementary Table 1). A total of 30.7% (261/852) of the isolates were phenotypically classified as MRSA. Of these, 98.5% (257/261 isolates) demonstrated resistance to both cefoxitin and oxacillin. Only three isolates were classified as susceptible to cefoxitin only, and one isolate to oxacillin only. The majority of the MRSA identified using in vitro phenotypic testing were detected in ST5 and ST8. Of the 261 MRSA isolates, 46 are from ST5 and 148 from ST8, representing 17.6 and 56.7% of the total number of MRSA isolates, respectively. These two MRSA lineages were consistently found throughout the duration of the study. MRSA was also detected in some members of ST45 (one isolate), ST72 (four isolates), ST97 (two isolates), and ST105 (36 isolates). In contrast, all ST30 isolates were susceptible to cefoxitin and oxacillin.

### A diverse, mobile pool of genetic variants is associated with AMR

**Fig. 2:**
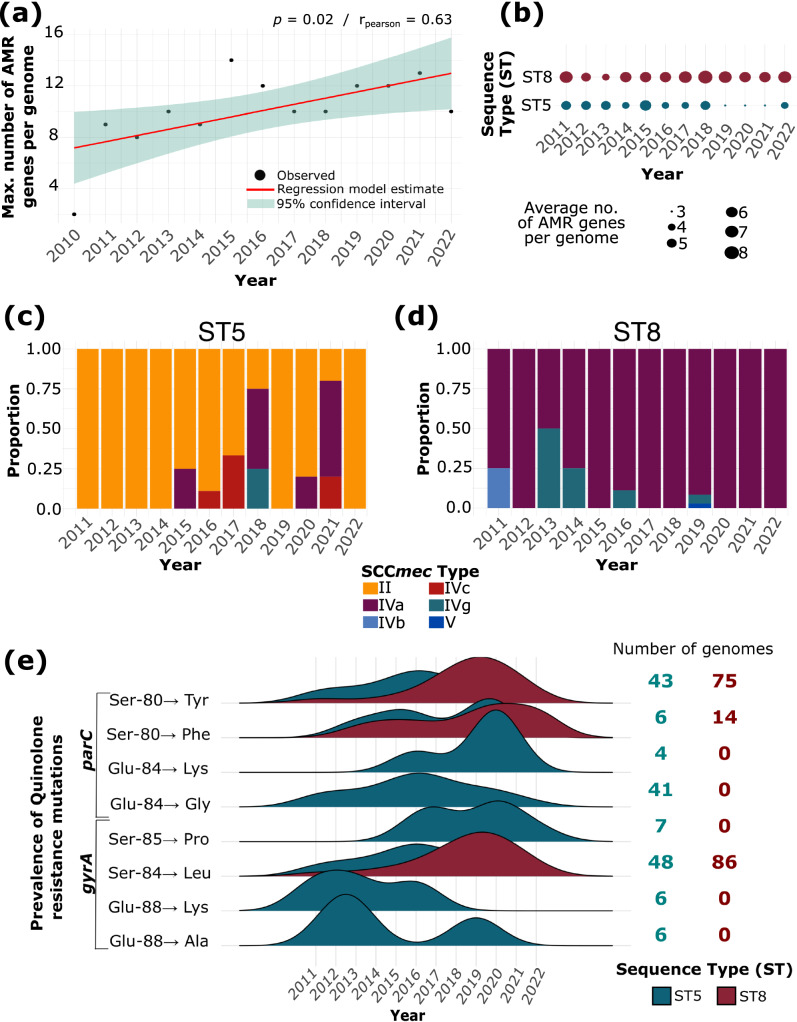
Antimicrobial resistance (AMR) in *S. aureus* isolates from bloodstream infection. **a** Linear regression model showing the maximum number of AMR genes per year. **b** Average number of AMR genes per genome in ST5 and ST8 per year. **c**, **d** Distribution of the different SCC*mec* types in **c** ST5 and **d** ST8 genomes per year. **e** Distribution of the different mutations in the genes *gyrA* and *parC* in ST5 and ST8 genomes per year. The number of genomes carrying a specific mutation is shown on the right and is colored by ST. Glu (Glutamic acid), Ala (Alanine), Lys (Lysine), Ser (Serine), Pro (Proline), Gly (Glycine), Phe (Phenylalanine), and Tyr (Tyrosine).

We identified a total of 24 distinct genes associated with resistance to at least nine antimicrobial classes (aminoglycosides, beta-lactams, folate pathway antagonists [trimethoprim], macrolides, fosfomycins, steroids [fusidic acid], lincosamide, pseudomonic acid [mupirocin], and tetracyclines) (Supplementary Table 4). On average, isolates carried a total of four AMR genes per genome (range: 0–14), conferring resistance to at least three different antimicrobial classes. We did not detect any AMR gene in 57 isolates. Only one isolate carried 14 AMR genes, and it belonged to ST5/BAPS14 from 2014. There was a significant increase in the maximum number of AMR genes per genome per year (*p* = 0.02, *r*_Pearson_ = 0.63; Fig. 2a).

We sought to characterize the mobile genetic elements that are associated with AMR. We detected the presence of six structural types of the chromosomal cassette SCC*mec*^21^, which carries the *mecA* gene encoding a modified penicillin-binding protein (PBP2a) with low affinity to most beta-lactams^22^. In total, we identified 260 genomes (30.5%) carrying the SCC*mec* (Supplementary Table 1). We also detected an average of three plasmids per genome (range: 0–7), with genomes belonging to ST8 and ST105 having the highest number of plasmids (Supplementary Table 5). A total of 24 types of plasmid replicon (Rep) proteins were present. While the closest plasmid database match of most predicted plasmids was *S. aureus*, there were three, two, and one genome/s carrying plasmids that were most closely related to *Listeria innocua, Streptococcus pyogenes*, and *Escherichia coli*, respectively. The majority of the reconstructed predicted plasmids (1,547/2,225 or 69.5%) were non-mobilizable, while the rest were mobilizable or conjugative. A total of 343 genomes harbored plasmids associated with 1–7 resistance genes per genome (Supplementary Table 5).

ST5 and ST8 contained genomes with multiple AMR genes throughout the duration of the study (Fig. 2b). The average number of AMR genes per genome was four in ST5 and six in ST8. However, we found that some AMR genes were more often detected in one ST than another. For example, the aminoglycoside nucleotidyltransferase genes *ant(4’)-Ia* and *ant(9)-Ia* were more frequently detected in ST5 (32 and 46 genomes, respectively) compared to ST8 (one and six genomes, respectively). In contrast, other aminoglycoside resistance genes were more commonly detected in ST8 genomes: *ant(6)-Ia* in 138 ST8 genomes and nine ST5 genomes, *aph(3’)-IIIa* in 159 ST8 genomes and ten ST5 genomes). The gene *ermA* (macrolide resistance) was also more frequently found in ST5 genomes, while the genes *dfrG* (folate pathway antagonists), *ermC*, *mphC*, *msrA* (macrolide resistance) and *sat4* (streptothricin resistance; N-glycoside antibiotics) were commonly detected in ST8 genomes (Supplementary Table 4). A total of 676 genomes harbored the *blaZ* gene, which encodes the beta-lactamase enzyme. Of these, 70 genomes belong to previously known STs and 21 genomes from the new STs described in this study. We identified five genomes carrying two copies of *blaZ* (three genomes from ST72, one from ST840, and one from ST582). *blaZ* was commonly detected in ST5 (present in 83/132 genomes) and ST8 (present in 200/225 genomes). Each ST carried different sets of SCC*mec* types (Fig. 2c, d). The most common SCC*mec* type in ST5 was type II, which was present in 35/132 or 26.5% of ST5 genomes. In ST8, SCC*mec* type IVa was the most common (present in 143/225 or 63.6% of the genomes). Both types were present in all 12 years of sampling. Four other SCC*mec* types appeared sporadically over the years in both STs. We also note that of the three isolates that phenotypically tested as susceptible to cefoxitin, two did not carry SCC*mec* while one had SCC*mec* type IVa. The one isolate that phenotypically tested as susceptible to oxacillin only was SCC*mec*-negative.

We screened both STs for the presence of specific mutations that are associated with resistance to fluoroquinolones (Fig. 2e and Supplementary Table 4). These mutations are found in the quinolone resistance-determining regions (QRDRs) of the chromosomal genes *gyrA* and *parC* that encode the DNA gyrase subunit A and DNA topoisomerase IV, respectively^23,24^. We detected eight mutations that are known to be associated with fluoroquinolone resistance. Throughout our sampling period, the prevalence of these mutations varied between the ST5 and ST8. The most common were the Ser → Tyr on codon 80 of *parC* and the Ser → Leu on codon 84 on *gyrA*. Both mutations were detected in genomes of ST5 and ST8, but their prevalence varied between them. The increase in frequency of the two mutations as well as in Ser → Phe on codon 80 in *parC* appears to correspond to the time when ST8 started to expand in the population (Fig. 1b).

Overall, the *S. aureus* population from bloodstream infections carries a highly variable accessory genome, consisting of numerous genes associated with AMR as well as mobile genetic elements such as chromosomal cassettes and plasmids. A summary of the temporal distribution of these genetic elements is presented in Supplementary Fig. 3.

### Virulence gene content varies between lineages

**Fig. 3:**
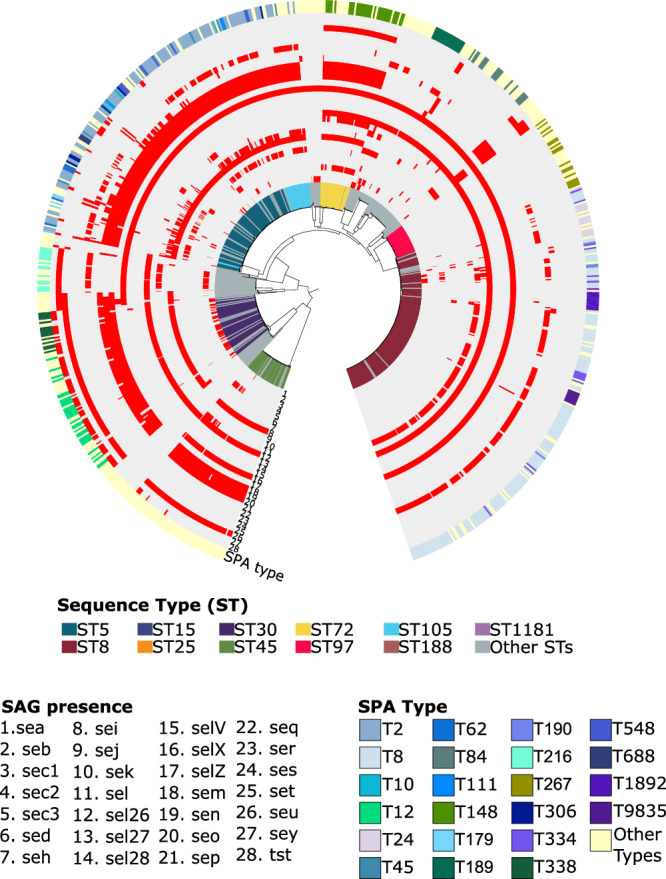
Phylogenetic distribution and diversity of virulence genes. The phylogenetic tree is identical to that in Fig. 1a. The colored lines extending out of the branches in the tree represent the ST. The outer rings numbered 1–28 represent the 28 SAg genes detected in the genomes. Red blocks indicate the presence of the SAg gene. The outermost ring shows the presence and types of the *spa* gene detected in the genomes. The gene names of SAg and the *spa* types are listed below the tree.

The bloodstream-associated *S. aureus* population harbored a wide variety of genes encoding virulence factors. We detected a total of 28 genes that encode superantigens (SAg), which comprise a large family of potent immunostimulatory exotoxins that cause T cell proliferation and massive cytokine release^25,26^ (Fig. 3 and Supplementary Table 4). Each genome carried five SAg genes (range: 0–13) on average. The genome harboring the highest number of SAg genes (*n* = 13) belonged to the ST840/BAPS14 sampled in 2011. The most frequently detected SAg genes were *selX* and *sel26*, which were found in 86.7% (739/852) and 74.2% (632/852) of the genomes, respectively. Only one genome (ST291/BAPS8 sampled in 2016) did not carry any SAg gene. We also observed that there were specific SAg genes that were often found together: *sel26* and *selX* in 153 genomes and *sek, sel26, selX*, and *seq* in 145 genomes. The set of SAg genes differed between ST5 and ST8, with *sed, sej, sep*, and *ser* present in ST5, and *sek* and *seq* in ST8.

We screened for the presence of the gene that encodes the staphylococcus protein A (SpA), which can trigger B cell proliferation^27^. The *spa* gene has been widely used for epidemiological surveillance of *S. aureus* because of its highly variable and repetitive regions^28^. We identified a total of 222 different *spa* types (Supplementary Table 4). Ten types (T8, T2, T12, T148, T189, T267, T84, T216, T334, and T338) were the most prevalent in the population and together were detected in 49.2% (419/852) of the genomes (Supplementary Fig. 4). A total of 29 and 32 different *spa* types were detected among ST5 and ST8 population, respectively. Type T2 is most common in ST5 (74/132 genomes) and type T8 in ST8 (146/225 genomes). Overall, the distribution and diversity of genes encoding SAg and SpA varied widely between the two lineages and throughout the sampling period.

### Independent acquisitions of SCCmec and AMR determinants in ST5 and ST8

**Fig. 4:**
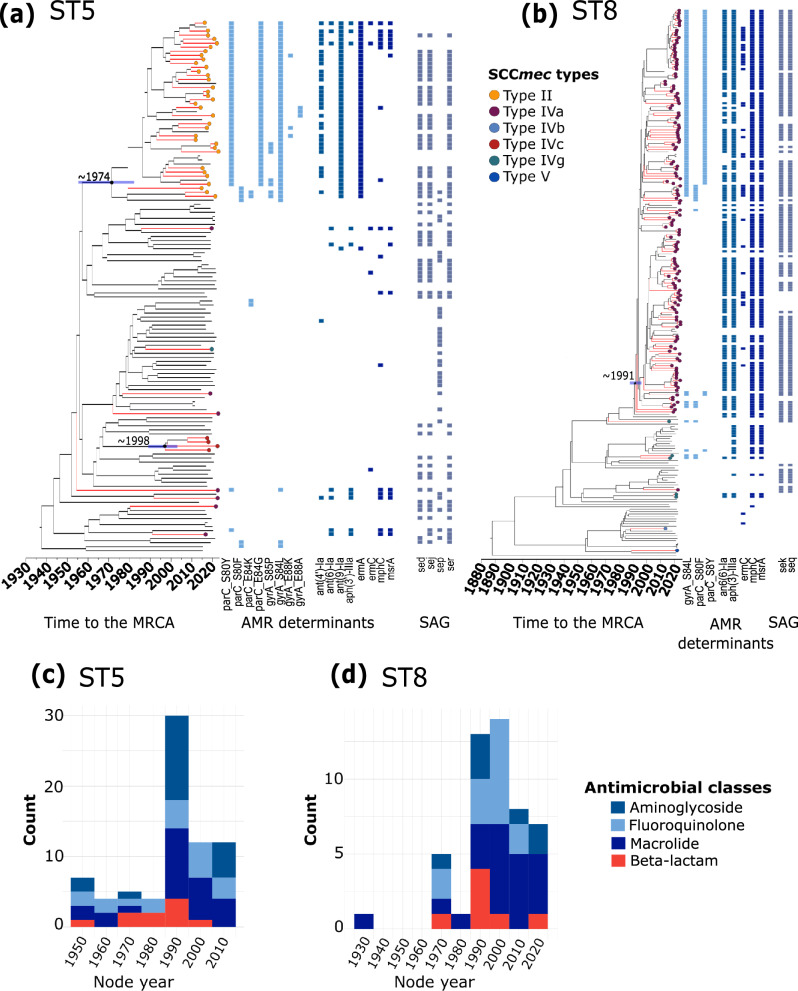
Molecular dating of ST5 and ST8. **a**, **b** Bayesian maximum clade credibility time-calibrated phylogenies of **a** ST5 and **b** ST8 based on non-recombining regions of the core genome alignment. Branches colored in red represent genomes carrying SCC*mec* elements. The SCC*mec* types are represented by the colored dots at the tip of the branches. The divergence date (median estimate with 95% highest posterior density dates) is indicated as a horizontal blue line on the tree. For visual clarity, only the relevant divergence dates related to SCC*mec* acquisition are shown. Colored squares represent the presence of a mutation or genes associated with AMR and superantigens (SAg). **c**, **d** Condensed version of panels **a** and **b** showing the emergence of AMR classes as a function of the age of nodes when resistance mutations or genes were inferred to have emerged in **c** ST5 and **d** ST8.

We next sought to estimate the timing of AMR acquisition events in ST5 and ST8 using time-calibrated phylogenies. First, using a recombination-free alignment of core genome SNPs, we determined the presence of a temporal signal in each lineage, i.e., there is a sufficient genetic change between sampling times to reconstruct a statistical relationship between genetic divergence and time. Using Bactdating^29^, we observed a positive relationship between dates of isolation and root-to-tip distances (ST5: *R*^2^ = 0.17, *p* < 1.00 × 10^-4^ and ST8: *R*^2^ = 0.16, *p* < 1.00 × 10^-4^), indicating a strong molecular clock signal (Supplementary Fig. 5a, b). The Deviance Information Criterion (DIC) values obtained comparing the original result of Bactdating with ten randomized runs confirmed the significance of the temporal signal (Supplementary Fig. 6 and Supplementary Tables 6, 8). Given the strong temporal structure for both STs, we then performed a dated coalescent phylogenetic analysis (Fig. 4a, b and Supplementary Fig. 5c, d). We estimated that the time to the most recent common ancestor (tMRCA) of ST5 was ~1937 (95% highest posterior density (HPD) intervals: 1909–1956), while the tMRCA of ST8 was ~1886 (95% HPD intervals: 1863–1906).

Our time-calibrated phylogenies revealed independent acquisition events of AMR determinants at different times in ST5 and ST8. Within ST5, a cluster consisting of 45 genomes acquired the SCC*mec* type II in 1974 (95% HPD intervals: 1956–1983) (Fig. 4a). Genomes comprising this cluster also harbored other AMR determinants: *ant(4’)-Ia*, *ant(9)-Ia*, *ermA*, mutations on *parC* (S80Y, E84G), and *gyrA* (S84L). Other SCC*mec* types (Iva, IVc, Ivg) were acquired by other ST5 lineages in at least eight independent events. Within ST8, a cluster consisting of 142 genomes harboring the SCC*mec* type IVa dates its tMRCA to 1991 (95% HPD intervals: 1987–1995). This cluster contains genomes carrying the AMR genes *ant(6)-Ia*, *aph(3)-IIIa*, *mphC*, and *msrA*. Half of the genomes within this cluster contain mutations in *gyrA* (S84L) and *parC* (S8Y), which were different from those found in ST5 genomes carrying the SCC*mec* type II. Summarizing the timing of all acquisition events of resistance determinants to aminoglycosides, fluoroquinolones, macrolides, and beta-lactams, we found the acquisition of AMR determinants started in the 1950s and 1930s in ST5 and ST8, respectively. For both STs, AMR acquisitions peaked in the 1990s and since then have declined (Fig. 4c, d).

**Fig. 5:**
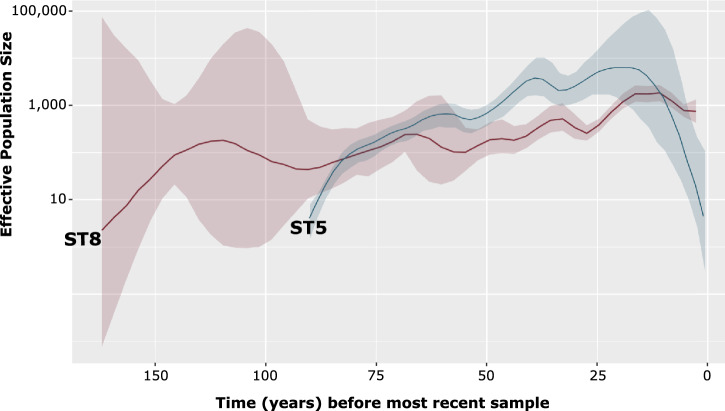
Bayesian skygrowth plot showing changes in effective population size (Ne) over time of ST5 and ST8. Median is represented by a solid line and 95% confidence intervals are represented by the shaded areas around the median.

We estimated the effective population sizes (Ne) of ST5 and ST8 in the population and how they have changed over time (Fig. 5). Overall, the two STs exhibited fluctuating demographies since their tMRCA, with the peaks and rapid increase coinciding with the times of acquisition events of specific AMR determinants. Approximately 80 years ago (1942), ST5 overtook ST8 in the population and continued to expand until 2007. Subsequently, ST5 rapidly declined, and ST8 became more frequent around 2015. The population dynamics of ST5 and ST8 may be partly attributed to differences in their recombination rates, which can influence the adaptive capacity of bacterial pathogens^30^. In ST5, we estimated the mean number of bases that experienced recombination was 156 bp (range: 0–1217 bp), r/m of 1.35 (range: 0–12), rho/theta of 0.026 (range: 0–0.875). In ST8, the mean number of bases that experienced recombination was 45 bp (range: 0–836), r/m of 1.47 (range: 0–175), and rho/theta of 0.015 (range: 0–0.4). The r/m parameter represents the ratio of diversity introduced by recombination and mutation, while rho/theta represents the relative rates of recombination and point mutation on a branch.

## Discussion

Population-level, multi-year surveillance of *S. aureus* genomes is a powerful approach to elucidating the genetic and epidemiologic features of strains and temporal trends in AMR. Here, we characterized the circulating lineages of *S. aureus* from bloodstream infections from a single hospital and the contribution of individual lineages to resistance over time. We highlight two major findings. First, *S. aureus* from bloodstream infections consists of genetically diverse co-circulating lineages. The population carries a remarkably wide array of genetic determinants associated with AMR (both acquired genes and mutations) and virulence. Second, the two lineages ST5 and ST8 exhibit fluctuating frequencies in the population and unique histories of AMR and SCC*mec* acquisition. Around 2015, ST8 showed evidence of surpassing ST5, which may have influenced the overall pool of genomic variants existing across the entire population.

*S. aureus* is characterized by the presence of numerous genetically distinct clonal lineages, but only a few have proliferated worldwide^12^. In bloodstream infections, higher frequencies of ST5 and ST8 has been previously reported^31–33^. These two lineages belong to the most prominent clonal complexes of *S. aureus* (CC5 and CC8, respectively) associated with the rapid dissemination of methicillin and multidrug resistance in both hospital and community settings^34,35^. Our results are consistent with previous reports of MRSA from bloodstream infections in other parts of the United States. In Pennsylvania (2018–2019), the most frequently detected were CC5 and CC8, while CC30, CC72, and CC78 were present at lower frequencies^36^. In Minnesota (2015), common MRSA clones were CC5 and CC8, but there were no MRSA isolates in CC45 or CC30^37^. In large-scale population genomic studies from other parts of the world, CC5 and CC8 are also prevalent in MRSA from bloodstream infections, but other lineages are also major contributors. In a study from Argentina, Bolivia, Brazil, Paraguay, and Uruguay, CC5, CC8, and CC30 were common in 2019^38^. Lineages CC5, CC8, and CC22 were dominant in Spain from 1990 to 2019^39^. In England, CC22 was dominant in 2012–2013, but CC5 and CC8 were found at lower frequencies^40^. In Japan, CC5 and CC1 were frequently detected, while CC8 was less common from 2019 to 2021^41^. In comparison, our study showed that CC30, CC45, and CC72 were frequently detected in MSSA but not MRSA, while CC1 and CC22 were rarely found. Our study shows that even within a single hospital, we can observe demographical patterns of major ST/CCs similar to those found in country-wide and global geographical scales. Altogether, our study and these previous investigations reveal that ST5/CC5 and ST8/CC8 continue to be major causal agents implicated in the persistence of bloodstream infections and as drivers of AMR spread worldwide. Continuous surveillance is critical to monitor dissemination between hospitals, regions, and countries, as well as any evidence of changing trends of resistance in these major clones. Such information may be particularly useful in developing effective control strategies for clinical treatment and public health interventions.

Our findings are also consistent with previous phylogenetic analyses of ST5/CC5 and ST8/CC8 (regardless of source) that revealed their early origins^34,42,43^, even before the emergence of MRSA in the 1960s^44^. The tMRCA for CC5-MRSA is estimated to be 1938, which was followed by expansions of two sublineages in the early 1960s and early 1970s in the American continents^45^. Community-acquired ST8 is estimated to have emerged in Central Europe in the mid-19th century and was exported to North America in the early 20th century^46^. The gradual replacement of ST5 by ST8 in the New Hampshire bloodstream population in recent years also mirrors findings of a decline in the prevalence of ST5 in other countries. In Colombia and Japan, ST5 was outcompeted by ST8^31,47^, while in Chile, ST5 was outcompeted by ST105^48^. ST764 and ST72 replaced ST5 in recent periods in China and South Korea, respectively^49,50^. The rise and fall of epidemic clones of *S. aureus*^34^ may reflect a scenario where a high number of co-circulating *S. aureus* and MRSA strains are in constant competition with each other within the confines of the prevailing environment. The factors determining the success or failure of individual clones are yet to be fully understood. Environmental changes, e.g., changes in the overall use and prescription rates of antibiotics in society, certainly play a role. However, we do not have this information in New Hampshire to make such inferences. Nonetheless, it is notable that there is greater genomic heterogeneity in ST5 in terms of its *spa* types, SAg genes, and SCC*mec* elements, which was also observed in genomic surveillance studies in China^51^ and southeast Austria^52^. Interestingly, the observed shift in the population structure in southeast Austria was due to the competition between two ST5 variants, which led to the replacement of ST5-SCC*mec* type I with ST5-SCC*mec* type II. Hence, genetic variants within ST5 can lead to niche competition within the lineage and may partly explain the shifts in the prevalence of ST5 in our study. Evolutionary trade-offs between the carriage of AMR and virulence genes may also explain the varying clonal dynamics in *S. aureus*. For example, the *agr* (accessory gene regulator) quorum sensing system is a major regulator of virulence phenotypes in *S. aureus*^53^ and its transcription is repressed by nafcillin and linezolid treatment, possibly by interference of resistance^54^. The genetic background can also alleviate some of the trade-offs (exhibited by reduced fitness and growth) caused by individual resistance elements^53,55^. While much remains to be learned regarding the factors driving proliferation of *S. aureus* clones in invasive diseases, the role of Darwinian selection within the genome and clonal fitness are critical to understanding the expansion and increased frequency of a few lineages and the eventual replacement of prior circulating strains. The population dynamics of the two high-frequency lineages ST5 and ST8 in New Hampshire occurring over ten years may reflect undetected selective events and changing ecology in isolates causing human invasive infection.

Not only can ST5 and ST8 help shape the gene pool of the entire population, less common STs can act as a major reservoir of mobile genes and allelic variants that can be shared with the high-frequency lineages. Different strains of the same species can exhibit variable rates of horizontal gene transfer and recombination^56^, which can substantially contribute to lineage differences in accessory gene composition. Hence, there is the potential for hypervirulent and multidrug-resistant strains to emerge from the convergence of clinically relevant genes within the same genome^57^. Long-term genomic surveillance of isolates from invasive diseases enables us to monitor and predict emerging high-risk clones as well as the dissemination of AMR genes over time. In our dataset, ST105 (sister lineage to ST5 within BAPS cluster 14) is increasingly concerning because of its persistence in the past nine years as a source of methicillin resistance as well as other AMR genes.

The challenge of *S. aureus* as a life-threatening cause of bloodstream infections is largely due to its arsenal of virulence factors, such as exotoxins that cause host cell injury as well as other protein products that facilitate tissue adhesion and immune evasion^58^, resistance to multiple antibiotics that can lead to treatment failure and poorer outcome^59^, and ubiquitous occurrence as a colonizer^2,3^. Here, we highlight the potential for different lineages and strains to produce different combinations of SAg toxins. The diversity of SAgs facilitates binding to a large repertoire of variable-beta chains in the T cell receptor and thus contributes to the pathogenesis of invasive *S. aureus*^60^. SAg have been reported to elicit redundant and extensive human Vβ T cell receptors^61^, which may explain the distinct sets of different SAg genes in ST5 and ST8 genomes. The combination of SAg genes that distinguish different lineages may provide potential targets for novel drug development against specific high-risk clones that cause life-threatening infections. Moreover, AMR and SAg genes can also be captured by mobile genetic elements^62^, which encounter competition against each other and experience their own evolution distinct from the cell that carries them^63^. We can therefore consider these clinically relevant genes as having their own ecological niches consisting of the genomic neighborhood that best enhances their own proliferation^64^.

We acknowledge the limitations of our study. First, the number of isolates collected varied considerably per year. Our isolates came from a routine surveillance study, and therefore the number of isolates is greatly influenced by the number of patients from which samples were obtained from. Such imperfect sampling can obfuscate the level of genetic diversity existing in the population and the frequency of STs and CCs in any one year. Second, only one isolate was obtained per patient. Multiple lineages of a bacterial species can co-exist within a single individual, as a result of repeated or mixed infections^65,66^ as well as the continuous lineage diversification occurring within a host^67,68^. Third, we do not have information about the history of colonization and treatment status of the patients; hence, we cannot trace the source of their bloodstream infections (i.e., foreign indwelling devices; surgery, skin, and soft tissue infection; pneumoniae), which may influence the bacterial lineages that enter and circulate in the bloodstream. Lastly, as in any time-calibrated phylogenetic analysis, estimation of the tMRCA is made difficult with increasing time between the ancestor and the observed descendants as well as the range of diversity of missing descendants. Hence, the estimated times of the MRCA of ST5 and ST8 represent only an approximation and may vary with the inclusion of additional isolates. Despite these limitations, our large genomic dataset derived from a single hospital surveyed continuously for more than a decade provides an important baseline census of circulating diversity and AMR in *S. aureus* from bloodstream infections.

Our study highlights the importance of long-term genomic surveillance to understanding the epidemiological features of *S. aureus* clones in bloodstream infections, the ecology of resistance dissemination, and rare lineages that may act as reservoirs of mobile AMR genes.

## Methods

### Bacterial isolates

Archived isolates of *S. aureus* from 869 unique pediatric and adult patient bloodstream infections were included in this study (Supplementary Table 1). Isolates were obtained from clinical blood culture specimens submitted to the Department of Pathology and Laboratory Medicine at Dartmouth-Hitchcock Medical Center (DHMC), New Hampshire, USA, from December 2010–February 2022. The first significant blood culture isolates from each patient is routinely archived (freezer space permitting) in case of future need for patient care, epidemiologic, public health, or laboratory quality studies. Upon subculture, isolates were assigned a study number, and all patient identifiers were removed with only the date of collection and results of phenotypic antimicrobial susceptibility testing linked to the study number. Ethical approval was granted by the Committee for the Protection of Human Subjects of Dartmouth-Hitchcock Medical Center and Dartmouth College. This study protocol was deemed not to be human subjects research. Samples used in the study were subcultured bacterial isolates that had been archived in the routine course of clinical laboratory operations. No patient specimens were used, and patient-protected health information was not collected. Therefore, informed consent was not required.

All isolates were tested in vitro on a commercial automated broth microdilution testing platform (MicroScan Walkaway, Beckman Coulter, Inc., La Brea, CA) against a panel of antimicrobial agents, including cefoxitin (screening well), daptomycin, oxacillin, penicillin, and vancomycin. Clinical breakpoints were interpreted according to Clinical Laboratory Standards Institute (CLSI) guidelines^69^. Methicillin resistance was determined per the manufacturer’s guidelines and CLSI guidelines by growth in the cefoxitin screening well (>4 μg/mL) and/or by an oxacillin minimum inhibitory concentration (MIC) of >2 μg/mL. All isolates were stored in a DMSO solution at −80 °C.

### DNA extraction and whole genome sequencing

Isolates were subcultured from DMSO stocks in brain heart infusion broth (BD Difco, Franklin Lakes, New Jersey) at 37 °C for 24 h. DNA was extracted and purified using the Zymo Research QuickDNA Fungal/ Bacterial Miniprep Kit (Irvine, California) following the manufacturer’s protocol. We used a Qubit fluorometer (Invitrogen, Grand Island, New York) to measure DNA concentration. DNA libraries from isolates sampled from December 2010 to August 2018 were prepared using the RipTide High Throughput Rapid DNA Library Prep kit (iGenomX, Carlsbad, California) and sequenced as multiplexed libraries on the Illumina HiSeq platform operated per the manufacturer’s instructions. Sequencing resulted in 250-nt long paired-end reads. Sequencing was carried out at the University of New Hampshire Hubbard Center for Genome Studies (Durham, New Hampshire). DNA libraries from isolates sampled from September 2018 to February 2022 were prepared using the Illumina DNA Prep kit and IDT 10 bp UDI indices. DNA was sequenced as multiplexed libraries on the Illumina NextSeq 2000 platform operated per the manufacturer’s instructions at the SeqCenter (Pittsburgh, Pennsylvania). Sequencing resulted in 151-nt long paired-end reads. The change in the DNA library protocol and sequencing platform was due to the senior author moving their laboratory to a new location. The first 323 genomes of this dataset have been previously described by our group^32^.

### De novo genome assembly, sequence quality check, and annotation

Reads were assembled into contigs using the Shovill v.1.1.0 pipeline (https://github.com/tseemann/shovill) with the option --trim. We used the scaffolding and gap-filling programs SSPACE^70^ and GapFiller^71^ to improve the quality of the assemblies. Genome quality was assessed using the programs QUAST^72^ and CheckM^73^ (Supplementary Table 1). Genomes with <90% completeness and >5% contamination, as recommended by CheckM^73^ were excluded from downstream analysis. We also excluded assemblies with >300 contigs and an N50 <40,000 bp. Genomes were compared to the *S. aureus* reference genome NCTC 8325 (NCBI Accession number NC_007795.1) using the program FastANI v.1.32^74^ to confirm species identification. We used the >95% average nucleotide identity (ANI) threshold to define a species^74^. After filtering out the genomes with <95% sequence identity, low coverage, and of poor quality, a total of 852 genomes were used for all downstream analyses (Supplementary Table 1 and Supplementary Fig. 1). The resulting contigs were annotated using Prokka v.1.14.6^75^.

### Determination of the core and accessory genomes

We used Panaroo v.1.2.7^76^ to determine the core and accessory gene content of the entire population. We defined core genes as those present in ≥95% of the genomes (i.e., 809 genomes), whereas the accessory genes were genes present in ≥1% and <95% of the dataset (i.e., 43 genomes). Panaroo was ran on strict mode with the refinding genes option to identify potential genes that have been missed by the annotation software. We used the default distance of 5000 nucleotides in the --search_radius option and a value of 80% in the –refind_prop_match option. Sequence alignment was carried out using MAFFT^77^. We also ran Panaroo only among members of the sequence type (ST) 5 lineage and only among members of the ST8 lineage. For every pair of genomes, we measured the divergence in the accessory genome content as pairwise Jaccard distances based on the presence and absence of coding sequences. The Jaccard distance was calculated using the micropan v.2.1 package^78^ in R v.4.1^79^.

### Phylogenetic and population structure analysis

Using the concatenated alignment of the 2128 core genes, we extracted the single nucleotide polymorphisms (SNP) using SNP-site v.2.5.1^80^. The aligned core SNPs were used to build a maximum likelihood phylogenetic tree using IQ-TREE v.2.1.4^81^ with a generalized time reversible (GTR)^82^ model of nucleotide substitution and Gamma distribution of rate heterogeneity. Support at nodes was estimated using 100 bootstrap replicates implemented using the built-in ultrabootstrap -UFBoot2^83^ (Supplementary Table 7). We used the Bayesian hierarchical clustering algorithm fastBAPS v.1.0.6 (fast Bayesian Analysis of Population Structure) to partition the genomes into sequence clusters consisting of genetically similar individuals^20^. Finally, the sequence type (ST) was determined using the program mlst v.2.19.0 (https://github.com/tseemann/mlst), which extracts seven housekeeping genes (*arcC*, *aroE*, *glpF*, *gmk*, *pta*, *tpi*, and *yqiL*)^19^ from the sequence contigs and compares sequence variation against previously characterized STs in the *S. aureus* PubMLST database^84^.

### In silico detection of antimicrobial resistance and superantigen genes

We used AMRFinderPlus v.3.11.14^85^ to identify the presence of AMR genes and genes related to superantigens (SAg). We used the minimum thresholds of ≥ 60% for sequence coverage and ≥ 90% sequence identity for comparing query sequence with the curated Reference Gene Database of the National Center for Biotechnology Information (NCBI). The default and recommended thresholds in AMRfinderPlus are ≥ 50% for sequence coverage and ≥ 90% for sequence identity, which are sufficient to distinguish AMR genes, including partial hits^85^. We increased the coverage threshold to 60% following the methods in recent AMR detection studies (e.g., refs. ^86–88^). We screened for the presence and type of *mecA*-carrying staphylococcal chromosomal cassette (SCC*mec*) using the stand-alone tool staphopia-sccmec v.1.0.0^88^. The virulence gene *spa*, which encodes the Staphylococcal protein A, is one of the most common methods for genotyping *S. aureus*^28^. We typed the highly variable X region of the *spa* gene using SpaType v.0.3.3 (https://github.com/HCGB-IGTP/spaTyper).

### Plasmid reconstruction

We used the MOB-recon tool v.3.1.0 from the MOB-suite software^89,90^, which calculates the mash min-hashing genetic distances to reconstruct and type the plasmids from all assembled genomes. To determine if the predicted plasmids carried AMR or SAg genes, we used the fasta files generated from the predicted plasmids and re-annotated them on AMRFinderPlus^85^ using the same settings as described above.

### Estimating the date of clonal origin and effective population size

For each ST5 and ST8, we identified the SNPs in the core genome alignment using Snippy v.4.6.0 (https://github.com/tseemann/snippy) and mapped them to a reference genome using the option snippy-multi. We chose as reference genome the first isolate detected in each of the two lineages in order to examine the microevolutionary changes within each ST over time in our population. The ST5 genomes were mapped against the genome of isolate 12sab, which was sampled in January 2011 (Accession number SAMN16605097). The ST8 genomes were mapped against the genome of isolate 25sab, which was sampled in May 2011 (Accession number SAMN16605196). Recombination rates were calculated using Gubbins v.3.2.1^91^. Using the recombination-free phylogenies generated by Gubbins^91^ for each ST, we used BactDating v.1.1.1^29^ to carry out a root-to-tip linear regression analysis. We calculated the coefficient of determination (R2) to assess the significance of the temporal signal based on random permutations of sampling dates. We then used BactDating to estimate the dates of the most recent common ancestor. We carried out 10^6^ iterations, removed the first half as burn-in, and subsequently sampled every 100 iterations. To assess the significance of the molecular clock signal, we run BactDating ten times for each group with random dates assigned to each isolate (Supplementary Table 8). Date randomization was performed using lubridate v.1.9.2^92^ package in R v.4.1^79^. The ten BactDating runs were performed using the same number of iterations as described above, then we used the modelcompare function on BactDating to compute the Deviance Information Criterion (DIC) between the original run and each of the ten randomized runs. The DIC is a metric used to compare the fit and complexity of Bayesian models^93^. For the phylodynamic analysis, we ran the R package Skygrowth v. 0.3.1^94^ on the recombination-free phylogenies of ST5 and ST8. Skygrowth employs a Bayesian Gibbs-within-Metropolis Markov Chain Monte Carlo and fast maximum *a posteriori* algorithms to estimate the effective population size through time and growth rates of effective population size. We ran Skygrowth with the default mode of 100,000 iterations for each tree and a smoothing parameter of 10 exponential for the prior.

### Statistical analysis

We carried out all statistical analysis using the ggstatsplot v.0.9.4 package^95^ in R v.4.1^79^. We used Welch’s *t*-test to compare the following parameters: the number of accessory genes, antimicrobial resistance genes, antimicrobial classes, plasmids, and superantigens. The same statistical test was also applied to compare the number of accessory genes, number of plasmids and plasmid size, core SNP distances, and Jaccard accessory distances between ST5 and ST8. The student’s *t*-test and Pearson correlation coefficient were performed to assess the regression model estimate of the core SNP distance average, and the maximum number of different ST, and AMR genes over time. Results were considered significant when *p* < 0.05.

### Reporting summary

Further information on research design is available in the Nature Research Reporting Summary linked to this article.

### Supplementary information

Supplemental figures 1-6

Supplemental tables 1-8

REPORTING SUMMARY

## Data Availability

The dataset supporting the conclusions of this article is included within the article and its supplementary files. Genome sequence data of *S. aureus* isolates are available in the NCBI Sequence Read Archive under BioProject accession number PRJNA673382. BioSample accession numbers for each genome are listed in Supplementary Table 1.
