# Supplementary material for: Demographic fluctuations in bloodstream Staphylococcus aureus lineages configure the mobile gene pool and antimicrobial resistance
Source: NPJ Antimicrob Resist. 2024 May 7;2:14. doi: 10.1038/s44259-024-00032-9 (PMC11076216; doi:10.1038/s44259-024-00032-9)
Supplement: Supplementary file 1 — Supplemental figures 1-6 [file 44259_2024_32_MOESM1_ESM.pdf]

Demographic fluctuations in bloodstream *Staphylococcus aureus* lineages configure the mobile gene pool and antimicrobial resistance

Stephanie S.R. Souza, Joshua T. Smith, Michael M. Marcovici, Elissa M. Eckhardt, Nicole B. Hansel, Isabella W. Martin, Cheryl P. Andam

Supplementary information

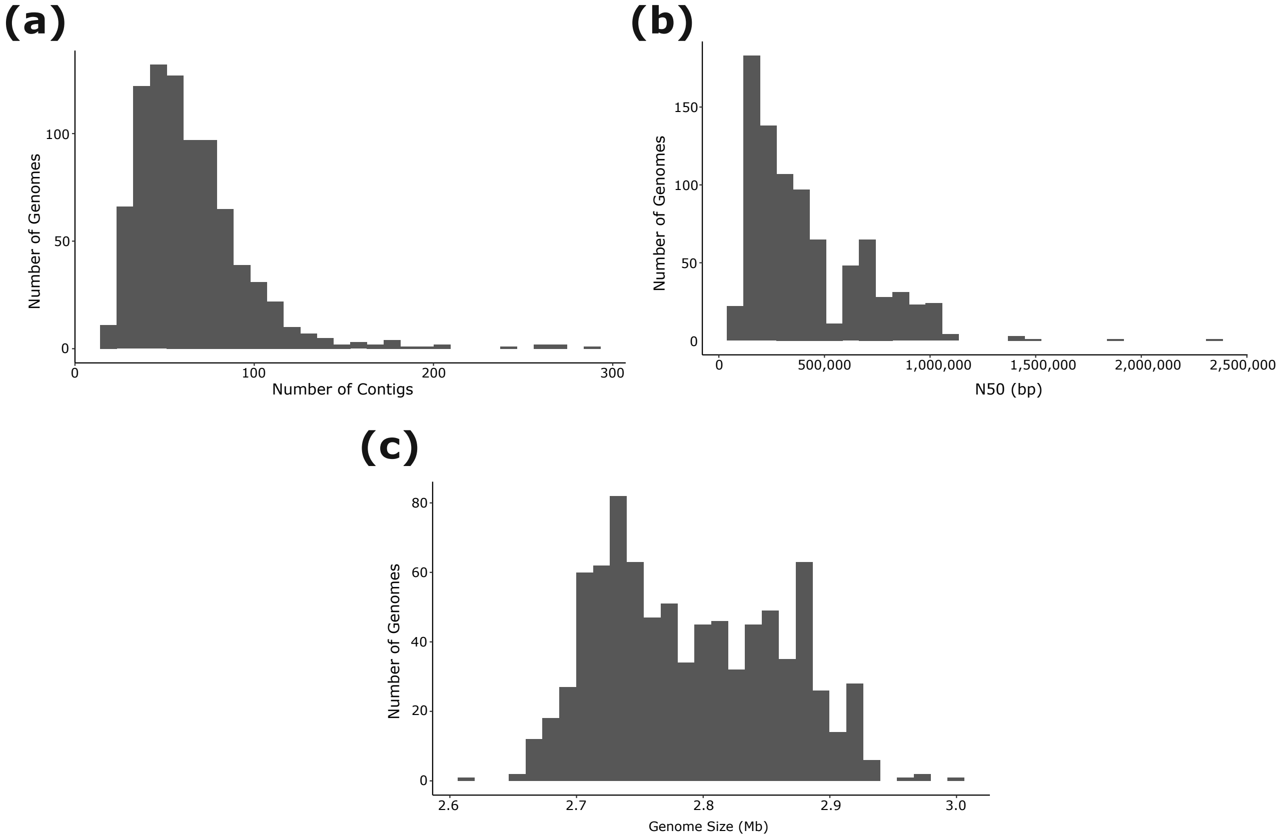

Supplementary Figure 1. Genome quality metrics of the 852 *S. aureus* isolates in this study. (a) number of contigs (b) N50 (c) genome size.

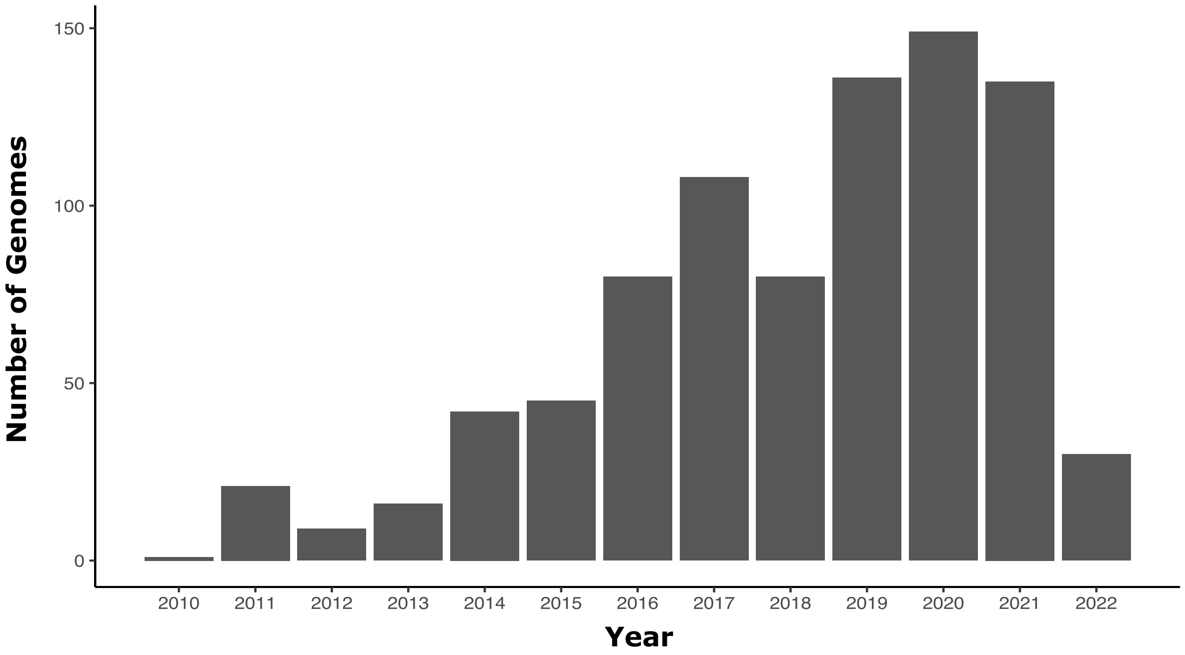

Supplementary Figure 2. Number of genomes included per year sampled throughout the duration of this study.

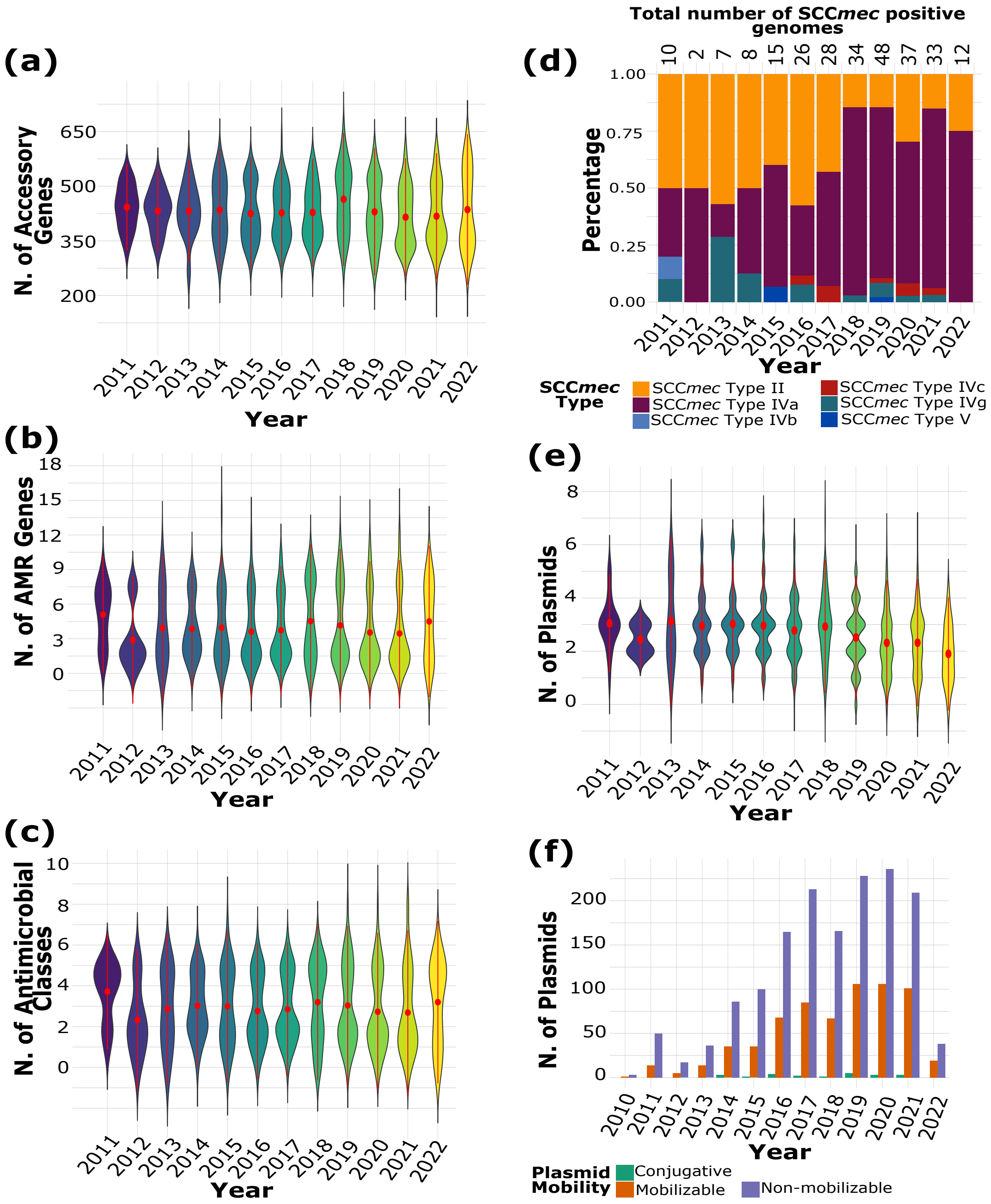

Supplementary Figure 3. Composition of the accessory genome of *S. aureus* (n = 852 isolates). (a-f) Yearly distribution of the (a) number of accessory genes, (b) antimicrobial resistance genes, (c) antimicrobial resistance classes, (d) SCC*mec* elements, (e) plasmids, and (f) types of plasmids according to predicted mobility. In the violin plots, the mean value is represented by the red dot, vertical line in the middle of the dot represents the standard deviation.

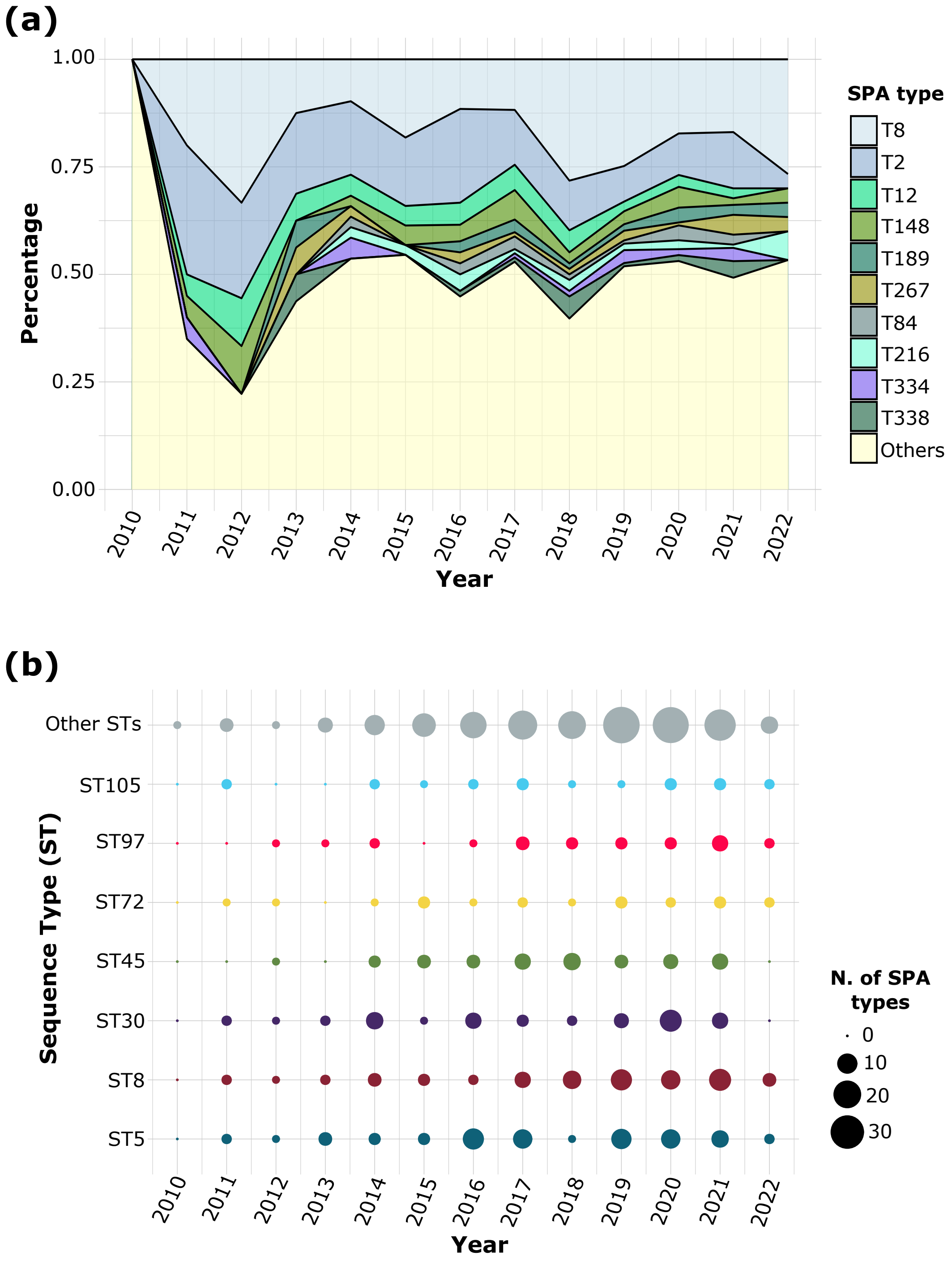

Supplementary Figure 4. Types and distribution of the *spa* gene that encodes *Staphylococcus* protein A (SpA) in the entire *S. aureus* population (n = 852 isolates). (a) Yearly distribution of SpA types throughout the study period. For visual clarity, only *spa* types represented by five or more genomes are shown. Other types are included in the category Others. (b) Yearly distribution of the number of different *spa* types among the most prevalent sequence types (ST) in the population. The size of the circles are proportional to the number of SpA types per ST per year.

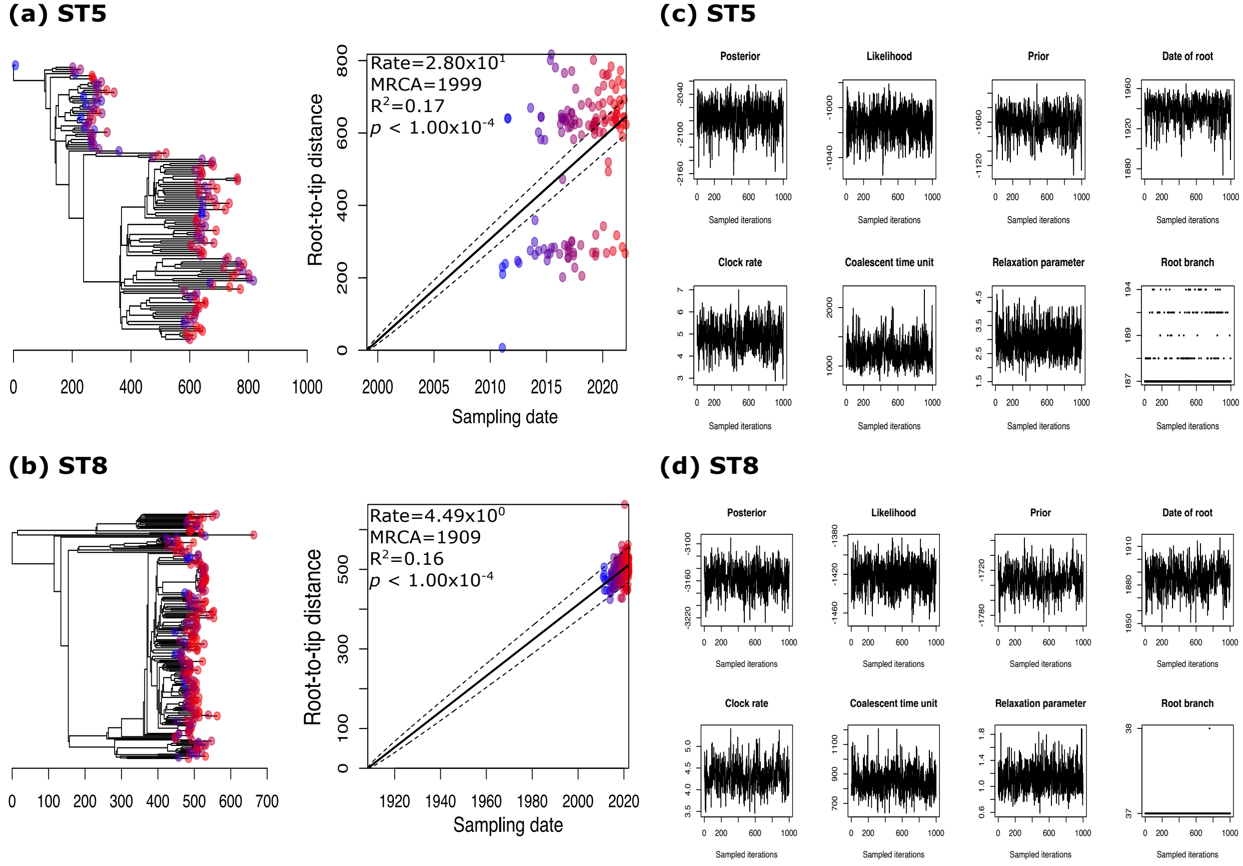

Supplementary Figure 5. Bactdating statistical tests and MCMC trace plots. (a,b) Initial rooted phylogeny and correlation test between date and root-to-tip distance within the phylogeny for ST5 (a) and ST8 (b). The colors of the dots correspond to the year of sampling, with the earliest years represented in blue and recent years in red. (c,b) Bactdating trace plots constructed by periodic sampling over the MCMC runs for ST5 (c) and ST8 (d).

(a) ST5

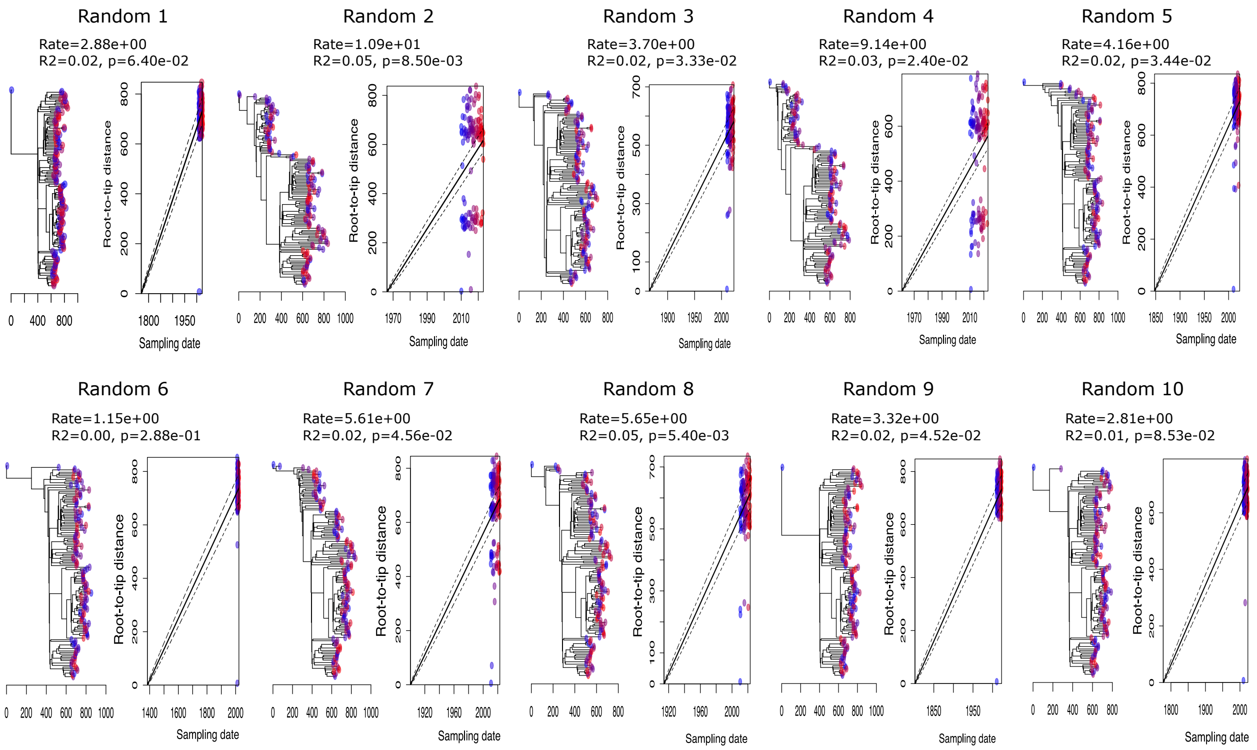

Random 1
Rate=2.88e+00
R2=0.02, p=6.40e-02
Random 2
Rate=1.09e+01
R2=0.05, p=8.50e-03
Random 3
Rate=3.70e+00
R2=0.02, p=3.33e-02
Random 4
Rate=9.14e+00
R2=0.03, p=2.40e-02
Random 5
Rate=4.16e+00
R2=0.02, p=3.44e-02
Random 6
Rate=1.15e+00
R2=0.00, p=2.88e-01
Random 7
Rate=5.61e+00
R2=0.02, p=4.56e-02
Random 8
Rate=5.65e+00
R2=0.05, p=5.40e-03
Random 9
Rate=3.32e+00
R2=0.02, p=4.52e-02
Random 10
Rate=2.81e+00
R2=0.01, p=8.53e-02
Root-to-tip distance
Sampling date

(b) ST8

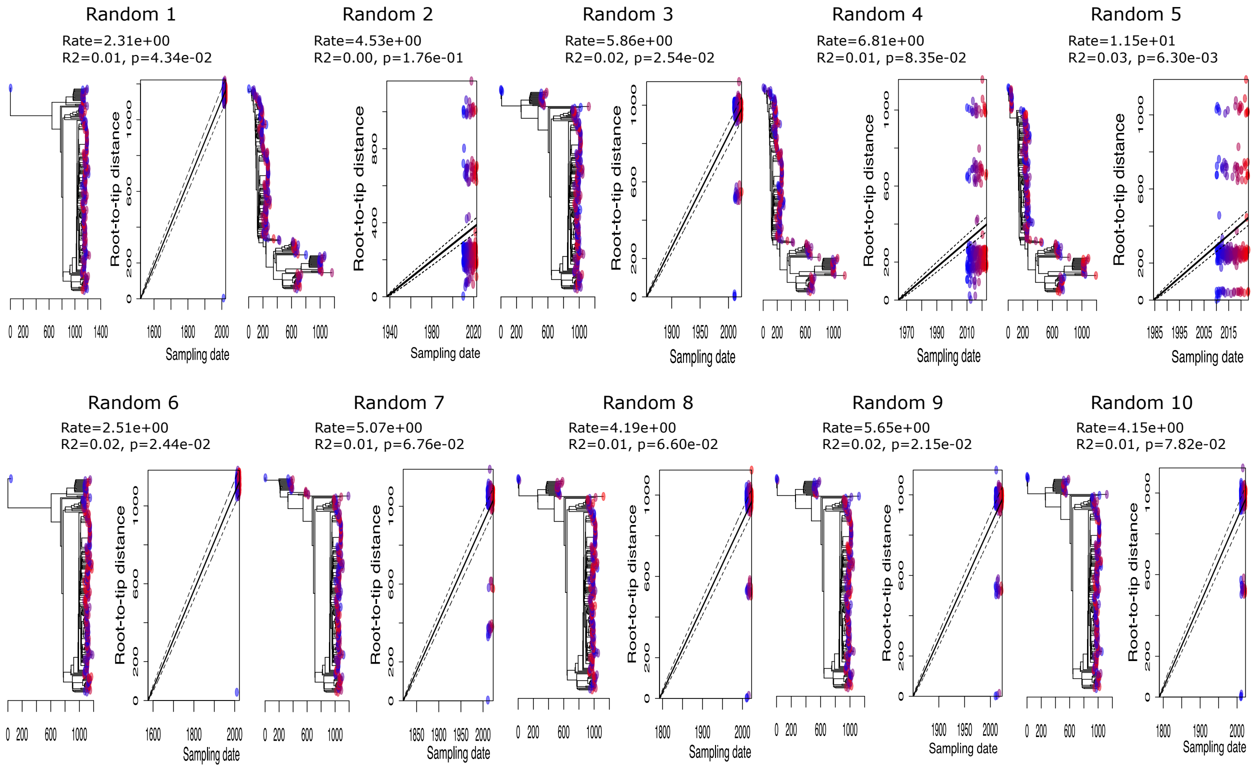

Random 1
Rate=2.31e+00
R2=0.01, p=4.34e-02
Random 2
Rate=4.53e+00
R2=0.00, p=1.76e-01
Random 3
Rate=5.86e+00
R2=0.02, p=2.54e-02
Random 4
Rate=6.81e+00
R2=0.01, p=8.35e-02
Random 5
Rate=1.15e+01
R2=0.03, p=6.30e-03
Random 6
Rate=2.51e+00
R2=0.02, p=2.44e-02
Random 7
Rate=5.07e+00
R2=0.01, p=6.76e-02
Random 8
Rate=4.19e+00
R2=0.01, p=6.60e-02
Random 9
Rate=5.65e+00
R2=0.02, p=2.15e-02
Random 10
Rate=4.15e+00
R2=0.01, p=7.82e-02
Root-to-tip distance
Sampling date

Supplementary Figure 6 (previous page). Results of the ten randomized runs of Bactdating for ST5 and ST8. Date randomization was performed using lubridate. The ten BactDating runs were performed using the same numbers of iterations as the original Bactdating run (Supplementary Figure 5). We then used the modelcompare function on BactDating to compute the Deviance Information Criterion (DIC) between the original run and each of the ten randomized runs.

Supplementary Table 1. Accession numbers, associated metadata, sequence types (ST), clonal complexes (CC), and sequence clusters inferred by fastBAPS (fast Bayesian Analysis of Population Structure) of the 852 *S. aureus* genomes in this study.

Supplementary Table 2. List of all genes detected using Panaroo in the pan-genome of 852 *S. aureus* from bloodstream infection**.**

Supplementary Table 3. List of the 29 newly assigned STs from this study.

Supplementary Table 4. Distribution of acquired genes and mutations associated with antimicrobial resistance (AMR) and the genes that encode superantigens (SAg), and *Staphylococcus* protein A.

Supplementary Table 5. Distribution and types of reconstructed plasmids determined by the MOB-recon program and the AMR genes associated with the plasmids.

Supplementary Table 6. Statistical significance of the temporal signal of ST5 and ST8 calculated using the Deviance Information Criterion (DIC) values implemented in lubricate. Ten randomized runs were carried out for each ST.

Supplementary Table 7. Tree file in nexus format of the core genome phylogeny of the 852 *S. aureus* isolates in Figure 1a.

Supplementary Table 8. Randomized dates in ST5 and ST8.
