# Supplementary material for: Demographic fluctuations in bloodstream Staphylococcus aureus lineages configure the mobile gene pool and antimicrobial resistance
Source: NPJ Antimicrob Resist. 2024 May 7;2:14. doi: 10.1038/s44259-024-00032-9 (PMC11076216; doi:10.1038/s44259-024-00032-9)
Supplement: Supplementary file 3 — REPORTING SUMMARY [file 44259_2024_32_MOESM3_ESM.pdf]

nature portfolio

Corresponding author(s): Andam

Last updated by author(s): Mar 1, 2024

Reporting Summary

Nature Portfolio wishes to improve the reproducibility of the work that we publish. This form provides structure for consistency and transparency in reporting. For further information on Nature Portfolio policies, see our Editorial Policies and the Editorial Policy Checklist.

Statistics

For all statistical analyses, confirm that the following items are present in the figure legend, table legend, main text, or Methods section.

n/a	Confirmed	
☐	☒	The exact sample size (*n*) for each experimental group/condition, given as a discrete number and unit of measurement
☐	☒	A statement on whether measurements were taken from distinct samples or whether the same sample was measured repeatedly
☐	☒	The statistical test(s) used AND whether they are one- or two-sided<br>*Only common tests should be described solely by name; describe more complex techniques in the Methods section.*
☐	☒	A description of all covariates tested
☐	☒	A description of any assumptions or corrections, such as tests of normality and adjustment for multiple comparisons
☐	☒	A full description of the statistical parameters including central tendency (e.g. means) or other basic estimates (e.g. regression coefficient) AND variation (e.g. standard deviation) or associated estimates of uncertainty (e.g. confidence intervals)
☐	☒	For null hypothesis testing, the test statistic (e.g. *F*, *t*, *r*) with confidence intervals, effect sizes, degrees of freedom and *P* value noted<br>*Give P values as exact values whenever suitable.*
☐	☒	For Bayesian analysis, information on the choice of priors and Markov chain Monte Carlo settings
☒	☐	For hierarchical and complex designs, identification of the appropriate level for tests and full reporting of outcomes
☐	☒	Estimates of effect sizes (e.g. Cohen's *d*, Pearson's *r*), indicating how they were calculated

Our web collection on statistics for biologists contains articles on many of the points above.

Software and code

Policy information about availability of computer code

Data collection

No software was used for data collection.

Data analysis

De novo genome assembly, sequence quality check, and annotation
Reads were assembled into contigs using the Shovill v.1.1.0 pipeline (https://github.com/tseemann/shovill) with the option --trim. We used the scaffolding and gap-filling programs SSPACE and GapFiller to improve the quality of the assemblies. Genome quality was assessed using the programs QUAST and CheckM (Supplementary Table 1). Genomes with < 90% completeness and > 5% contamination as recommended by CheckM were excluded from downstream analysis. We also excluded assemblies with > 300 contigs and an N50 < 40,000 bp. Genomes were compared to the S. aureus reference genome NCTC 8325 (NCBI Accession number NC_007795.1) using the program FastANI v.1.32 to confirm species identification. We used the > 95% average nucleotide identity (ANI) threshold to define a species. After filtering out the genomes with < 95% sequence identity, low coverage and of poor quality, a total of 852 genomes were used for all downstream analyses (Supplementary Table 1 and Supplementary Figure 1). The resulting contigs were annotated using Prokka v.1.14.6.

Determination of the core and accessory genomes
We used Panaroo v.1.2.7 to determine the core and accessory gene content of the entire population. We defined core genes as those present in ≥ 95% of the genomes (i.e., 809 genomes), whereas the accessory genes were genes present in ≥ 1% and < 95% of the dataset (i.e., 43 genomes). Panaroo was ran on strict mode with the refinding genes option to identify potential genes that have been missed by the annotation software. We used the default distance of 5,000 nucleotides in the --search_radius option and a value of 80% in the –refind_prop_match option. Sequence alignment was carried out using MAFFT. We also ran Panaroo only among members of the sequence type (ST) 5 lineage and only among members of the ST8 lineage. For every pair of genomes, we measured the divergence in the accessory genome content as pairwise Jaccard distances based on the presence and absence of coding sequences. The Jaccard distance was calculated using the micropan v.2.1 package 78 in R v.4.1.

Phylogenetic and population structure analysis
Using the concatenated alignment of the 2,128 core genes, we extracted the single nucleotide polymorphisms (SNP) using SNP-site v.2.5.1. The aligned core SNPs were used to build a maximum likelihood phylogenetic tree using IQ-TREE v.2.1.4 with a generalized time reversible (GTR) model of nucleotide substitution and Gamma distribution of rate heterogeneity. Support at nodes was estimated using 100 bootstrap replicates implemented using the built-in ultrabootstrap -UFBoot2 (Supplementary Table 7). We used the Bayesian hierarchical clustering algorithm fastBAPS v.1.0.6 (fast Bayesian Analysis of Population Structure) to partition the genomes into sequence clusters consisting of genetically similar individuals. Finally, the sequence type (ST) was determined using the program mlst v.2.19.0 (https://github.com/tseemann/mlst), which extracts seven housekeeping genes (arcC, aroE, glpF, gmk, pta, tpi, and yqiL) from the sequence contigs and compares sequence variation against previously characterized STs in the S. aureus PubMLST database.

In silico detection of antimicrobial resistance and superantigen genes
We used AMRFinderPlus v.3.11.14 to identify the presence of AMR genes and genes related to superantigens (SAg). We used the minimum thresholds of >= 60% for sequence coverage and >=90% sequence identity for comparing query sequence with the curated Reference Gene Database of the National Center for Biotechnology Information (NCBI). The default and recommended thresholds in AMRfinderPlus are >=50% for sequence coverage and >= 90% sequence identity, which are sufficient to distinguish AMR genes including partial hits. We increased the coverage threshold to 60% following the methods in recent AMR detection studies. We screened for the presence and type of mecA-carrying staphylococcal chromosomal cassette (SCCmec) using the stand-alone tool staphopia-sccmec v.1.0.0. The virulence gene spa, which encodes the Staphylococcal protein A, is one of the most common methods for genotyping S. aureus. We typed the highly variable X region of the spa gene using SpaType v.0.3.3 (https://github.com/HCGB-IGTP/spaTyper).

Plasmid reconstruction
We used the MOB-recon tool v.3.1.0 from the MOB-suite software, which calculates the mash min-hashing genetic distances to reconstruct and type the plasmids from all assembled genomes. To determine if the predicted plasmids carried AMR or SAg genes, we used the fasta files generated from the predicted plasmids and re-annotated them on AMRFinderPlus using the same settings as described above.

Estimating the date of clonal origin and effective population size
For each ST5 and ST8, we identified the SNPs in the core genome alignment using Snippy v.4.6.0 (https://github.com/tseemann/snippy) and mapped them to a reference genome using the option snippy-multi. We choose as reference genome the first isolate detected in each of the two lineages in order to examine the microevolutionary changes within each ST over time in our population. The ST5 genomes were mapped against the genome of isolate 12sab, which was sampled in January 2011 (Accession number SAMN16605097). The ST8 genomes were mapped against the genome of isolate 25sab, which was sampled in May 2011 (Accession number SAMN16605196). Recombination rates were calculated using Gubbins v.3.2.1. Using the recombination-free phylogenies generated by Gubbins for each ST, we used BactDating v.1.1.1 to carry out a root-to-tip linear regression analysis. We calculated the coefficient of determination (R2) to assess the significance of the temporal signal based on random permutations of sampling dates. We then used BactDating to estimate the dates of the most recent common ancestor. We carried out 106 iterations, removed the first half as burn-in, and subsequently sampled every 100 iterations. To assess the significance of the molecular clock signal, we run BactDating ten times for each group with random dates assigned to each isolate (Supplementary Table 8). Date randomization was performed using lubridate v.1.9.2 package in R v.4.1.. The ten BactDating runs were performed using the same numbers of iterations as described above, then we used the modelcompare function on BactDating to compute the Deviance Information Criterion (DIC) between the original run and each of the ten randomized runs. The DIC is a metric used to compare the fit and complexity of Bayesian models. For the phylodynamic analysis, we ran the R package Skygrowth v. 0.3.1 on the recombination-free phylogenies of ST5 and ST8. Skygrowth employs a Bayesian Gibbs-within-Metropolis Markov Chain Monte Carlo and fast maximum a posteriori algorithms to estimate the effective population size through time and growth rates of effective population size. We ran Skygrowth with the default mode of 100,000 iterations for each tree and a smoothing parameter of 10 exponential for the prior.

Statistical analysis
We carried out all statistical analysis using the ggstatsplot v.0.9.4 package in R v.4.1. We used Welch's t-test to compare the following parameters: the number of accessory genes, antimicrobial resistance genes, antimicrobial classes, plasmids, and superantigens. The same statistical test was also applied to compare the number of accessory genes, number of plasmids and plasmid size, core SNP distances, and Jaccard accessory distances between ST5 and ST8. The student's t-test and Pearson correlation coefficient were performed to assess the regression model estimate of the core SNP distance average, the maximum number of different ST, and AMR genes over time. Results were considered significant when $p < 0.05$.

For manuscripts utilizing custom algorithms or software that are central to the research but not yet described in published literature, software must be made available to editors and reviewers. We strongly encourage code deposition in a community repository (e.g. GitHub). See the Nature Portfolio guidelines for submitting code & software for further information.

Data

Policy information about availability of data

All manuscripts must include a data availability statement. This statement should provide the following information, where applicable:

- Accession codes, unique identifiers, or web links for publicly available datasets
- A description of any restrictions on data availability
- For clinical datasets or third party data, please ensure that the statement adheres to our policy

The dataset supporting the conclusions of this article is included within the article and its supplementary files. Genome sequence data of S. aureus isolates are available in the NCBI Sequence Read Archive under BioProject accession number PRJNA673382. BioSample accession numbers for each genome are listed in Supplementary Table 1.

Research involving human participants, their data, or biological material

Policy information about studies with human participants or human data. See also policy information about sex, gender (identity/presentation), and sexual orientation and race, ethnicity and racism.

Reporting on sex and gender	Not applicable
Reporting on race, ethnicity, or other socially relevant groupings	Not applicable
Population characteristics	Not applicable
Recruitment	Not applicable
Ethics oversight	Ethical approval was granted by the Committee for the Protection of Human Subjects of Dartmouth-Hitchcock Medical Center and Dartmouth College. This study protocol was deemed not to be human subjects research. Samples used in the study were subcultured bacterial isolates that had been archived in the routine course of clinical laboratory operations. No patient specimens were used and patient protected health information was not collected. Therefore, informed consent was not required.

Note that full information on the approval of the study protocol must also be provided in the manuscript.

Field-specific reporting

Please select the one below that is the best fit for your research. If you are not sure, read the appropriate sections before making your selection.

☒ Life sciences ☐ Behavioural & social sciences ☐ Ecological, evolutionary & environmental sciences

For a reference copy of the document with all sections, see nature.com/documents/nr-reporting-summary-flat.pdf

Life sciences study design

All studies must disclose on these points even when the disclosure is negative.

Sample size	No sample size calculation was performed.<br>Sample size is based on the number of bacterial isolates from clinical animal specimens received by the Dartmouth-Hitchcock Medical Center.<br>The number of genomes analyzed is based on the number of high quality sequences obtained.
Data exclusions	No data were excluded from the analyses. All isolates collected and genomes sequenced were included.
Replication	Replication is not relevant because all genomes that were sequenced were included in the analyses.
Randomization	Randomization is not relevant because all genomes that were sequenced were included in the analyses.
Blinding	Blinding is not relevant because all bacterial isolates and genomes were included in the analyses.

Reporting for specific materials, systems and methods

We require information from authors about some types of materials, experimental systems and methods used in many studies. Here, indicate whether each material, system or method listed is relevant to your study. If you are not sure if a list item applies to your research, read the appropriate section before selecting a response.

Materials & experimental systems

n/a	Involved in the study
☒	☐ Antibodies
☒	☐ Eukaryotic cell lines
☒	☐ Palaeontology and archaeology
☒	☐ Animals and other organisms
☒	☐ Clinical data
☒	☐ Dual use research of concern
☒	☐ Plants

Methods

n/a	Involved in the study
☒	☐ ChIP-seq
☒	☐ Flow cytometry
☒	☐ MRI-based neuroimaging

Plants

Seed stocks	Not applicable
Novel plant genotypes	Not applicable
Authentication	Not applicable
